# A new simple and innovative technique for surgical drains fixation

**DOI:** 10.1093/jscr/rjad687

**Published:** 2023-12-28

**Authors:** Abeer Alsherawi, Fatima Saoud Al-Mohannadi, Mohamed Badie Ahmed

**Affiliations:** Plastic Surgery Department, Hamad General Hospital, Hamad Medical Corporation, Doha, Qatar; Plastic Surgery Department, Hamad General Hospital, Hamad Medical Corporation, Doha, Qatar; Plastic Surgery Department, Hamad General Hospital, Hamad Medical Corporation, Doha, Qatar; College of Medicine, QU Health, Qatar University, Doha, Qatar

**Keywords:** Surgical drain, Seroma, Technique, Complications

## Abstract

Seroma is a common complication post many plastic surgery procedures. To overcome this issue, drain insertion became a standard of care in many procedures. Existing methods for fixing the drain like the Roman sandal, purse string, and mesentery have limitations, including loosening and skin problems. A new, innovative, and efficient drain fixation technique is introduced in this paper. It involves using silk or similar non-absorbable sutures in a simple five-step process. This method ensures secure drain placement without undesirable outcomes. It avoids the need to force a knot over the skin, reducing the risk of skin damage or necrosis. In conclusion, the study introduces a straightforward, safe, and effective drain fixation method, reducing risks associated with fluid accumulation after surgery.

## Introduction

One of the most common post-surgical complications is the collection of fluids in dead spaces. This complication can be a burden to both the patient and the surgeon because it might lead to further adverse outcomes. Fluid accumulation can be quite painful and upsetting to the patient. In addition, it can be stressful to the surgeon since there is a possibility of developing infections and wound dehiscence, which sometimes require surgical debridement and drainage [1]. The best method to manage this complication is by prevention. This can be established by intraoperative drain insertion. However, it is important to have a proper technique of drain fixation for it to remain in place and serve its purpose. The security of the drain to the skin can be affected by the tightness of the surgical knot, the pulling forces on the drain and the surgeon’s technique [2]. Careful considerations include avoiding tube kinking or obstruction.

There are various methods of drain fixation proposed in the literature, one of them is the traditional Roman sandal. It involves placing a surgical knot next to the drain site followed by several secondary knots around the drain tube. Another method is by placing a purse string around the drain exit site. Furthermore, some surgeons preferer to use the mesentery method, where a drain is secured using adhesive tapes. Nevertheless, these methods have been argued to sometimes become loose or cause scaring and skin necrosis [3, 4].

The new drain fixation technique presented is innovative and efficient as well as easy to apply and remove without causing undesirable outcomes.

## Technique

For this simple technique we use silk or any other non-absorbable multifilament suture type, and it consists basically of five steps:

1. First, a simple stitch is taken in the skin 2–4 mm away from the drain, without knotting it.
2. The second step is very important, in which the surgeon holds the drain in one hand and both threads in the other hand, as in a preparation for a surgical knot, imagining that the drain is a thread, and the two silk threads as the other thread.
3. Now, the surgeon performs a simple surgical knot, turning both threads around the drain. We recommend 4 to 6 knots. The knots should be tight enough to fix the drain, but not too tight as it can block it. The tightness should be adjusted according to the softness of the drain material.
4. The surgeon should make sure to push all the loops tightly next to each other.
5. Finally, all of the loops should be secured with an extra knot that is made by the two threads only.

Demonstration video is available at the journal website.

## Discussion

This technique offers an easy, simple, fast, and elegant way to secure the surgical drain, without forcing a knot over the skin. It protects the skin from pressure necrosis or damage and keeps the drain in situ very securely for longer periods of time. We used this technique in our patients, without any complications like leakage, migration of the drain or skin necrosis. The removal of the drain is easy as there is no knot over the skin. Several surgical drain fixation techniques have been published and described in the literature [2–6]. However, our technique is different in terms of the steps and the neat outcome. In addition, it is very easy to learn and very smooth in its application. This technique could be used to fix any surgical drain and could be applied at any body site. We use it in our plastic surgery procedures, and it did not show any drawback when compared with other techniques in terms of infection, scarring, skin necrosis and slippage.

## Conclusion

In this paper, we introduce an uncomplicated, smooth, and safe technique for securing drains. It is straightforward to acquire, can remain securely in position for long periods of time, and does not pose any skin-related risks.

## Supplementary Material

drain_vid_rjad687
